# Role of Endothelial STAT3 in Cerebrovascular Function and Protection from Ischemic Brain Injury

**DOI:** 10.3390/ijms232012167

**Published:** 2022-10-12

**Authors:** Catherine M. Davis, Kristin Lyon-Scott, Elena V. Varlamov, Wenri H. Zhang, Nabil J. Alkayed

**Affiliations:** 1 Department of Anesthesiology & Perioperative Medicine, Oregon Health & Science University, 3181 S.W. Sam Jackson Pk. Rd., UHN-2, Portland, OR 97239-3098, USA; 2 Department of Medicine, Division of Endocrinology and Department of Neurological Surgery, Oregon Health & Science University, 3181 S.W. Sam Jackson Pk. Rd., UHN-2, Portland, OR 97239-3098, USA; 3 The Knight Cardiovascular Institute, Oregon Health & Science University, 3181 S.W. Sam Jackson Pk. Rd., UHN-2, Portland, OR 97239-3098, USA

**Keywords:** STAT3, endothelial cell, cerebral ischemia, BBB

## Abstract

STAT3 plays a protective role against ischemic brain injury; however, it is not clear which brain cell type mediates this effect, and by which mechanism. We tested the hypothesis that endothelial STAT3 contributes to protection from cerebral ischemia, by preserving cerebrovascular endothelial function and blood–brain barrier (BBB) integrity. The objective of this study was to determine the role of STAT3 in cerebrovascular endothelial cell (EC) survival and function, and its role in tissue outcome after cerebral ischemia. We found that in primary mouse brain microvascular ECs, STAT3 was constitutively active, and its phosphorylation was reduced by oxygen-glucose deprivation (OGD), recovering after re-oxygenation. STAT3 inhibition, using two mechanistically different pharmacological inhibitors, increased EC injury after OGD. The sub-lethal inhibition of STAT3 caused endothelial dysfunction, demonstrated by reduced nitric oxide release in response to acetylcholine and reduced barrier function of the endothelial monolayer. Finally, mice with reduced endothelial STAT3 (Tie2-Cre; STAT3^flox/wt^) sustained larger brain infarcts after middle cerebral artery occlusion (MCAO) compared to wild-type (WT) littermates. We conclude that STAT3 is vital to maintaining cerebrovascular integrity, playing a role in EC survival and function, and protection against cerebral ischemia. Endothelial STAT3 may serve as a potential target in preventing endothelial dysfunction after stroke.

## 1. Introduction

Signal Transducer and Activator of Transcription 3 (STAT3) plays a role in a wide range of biological processes in brain, including protection from cerebral ischemia [1,2,3,4]. The canonical model of activation requires phosphorylation of Janus kinases (JAKs), leading to tyrosine phosphorylation of STAT3, subsequent dimerization and translocation from the cytoplasm to the nucleus where it exerts its role as a transcription factor [5]. The most widely studied role for STAT3 is that of a survival factor; it protects a wide variety of cell types from pro-apoptotic stimuli [6,7,8]. In the rodent brain STAT3 is protective in stroke; pharmacological inhibition of STAT3, which is upregulated during reperfusion, results in increased infarct after middle cerebral artery occlusion (MCAO). Its neuroprotective role has been attributed to reduction in reactive oxygen species during reperfusion, and has been proposed to mediate the neuroprotective effects of estradiol, leptin, interleukin-10 and curcumin [1,2,3,9,10,11,12,13,14]. However, although global pharmacological inhibition of STAT3 is detrimental in stroke, activation of STAT3 in some cell types, specifically microglia, can result in detrimental outcomes following MCAO [15,16,17]. The cell-specific contribution of STAT3 to outcome following MCAO has not yet been determined for every cell type found in the brain, especially cerebrovascular endothelial cells (ECs).

Endothelial integrity and function are important determinants of stroke outcome. Cerebral ECs are a vital component of the microvasculature supplying the brain with oxygen and nutrients. Together with pericytes and astrocytes, ECs form the blood–brain barrier (BBB). While these cells act in concert, it is primarily the cohesiveness of the ECs that determines the integrity of this barrier. As such, ECs are the brain’s first line of defense against many insults. Failure of ECs to overcome insult leads to endothelial dysfunction and BBB dysfunction, which has been implicated in many central nervous system pathologies including stroke [18,19,20]. Cerebral endothelial injury following ischemia leads to increased permeability of the BBB and edema, contributing to post-ischemic brain injury [21,22]. Endothelial dysfunction is therefore a major contributor to both the development and outcome of stroke.

The aims of this study are to define the function of STAT3 in primary brain endothelial cells and its contribution to ischemic brain injury following MCAO. Since STAT3 is protective to many cell types, we hypothesized that STAT3 would protect cerebrovascular ECs from cell death and dysfunction, and that endothelial-specific attenuation of STAT3 would increase infarct size following MCAO.

## 2. Results

### 2.1. Endotheilal STAT3 Activation Is Regulated by OGD

**Figure 1:**
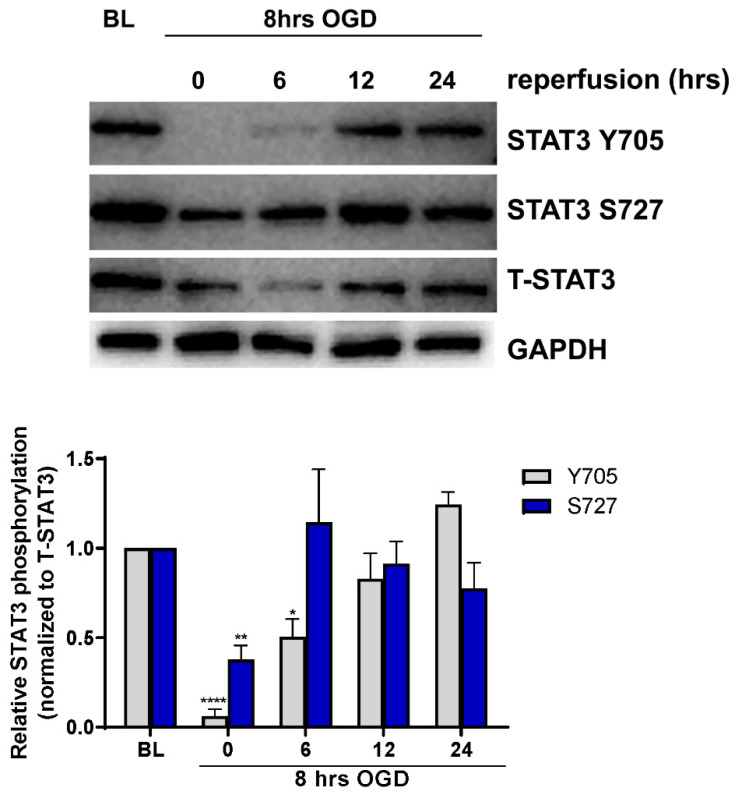
Regulation of STAT3 phosphorylation by OGD. ECs were subjected to 8 h of OGD followed by return to normal glucose and oxygen conditions for 0, 6, 12 and 24 h before being harvested for Western blot analysis. Blots were probed for phospho-STAT3 (Y705), phospo-STAT3 (S727), total STAT3 (T-STAT3) and GAPDH. Phosphorylation following OGD was compared to non-OGD controls (baseline; BL). Phosphorylation on both Y705 and S727 residues was reduced following OGD, returning to baseline levels during reperfusion. *n* = 7; * *p* < 0.05, ** *p* < 0.01, **** *p* < 0.0001, 2-way ANOVA. Data are presented as mean ± SEM.

To determine whether ischemia alters STAT3 activation in cerebral microvascular ECs, primary cultured ECs were subjected to ischemia-like injury induced by incubation under oxygen-glucose deprivation (OGD) for 8 h, followed by incubation in glucose-containing medium under normoxic conditions (reoxygenation) for 0, 6, 12 and 24 h. Cells were harvested and analyzed for STAT3 activation by measuring phosphorylation on both tyrosine 705 (Y705) and serine 727 (S727) residues using Western blot (Figure 1). Under baseline conditions, phosphorylation of both Y705 and S727 residues was observed. Phosphorylation of Y705 was significantly reduced to 5 ± 3.03% of its baseline level immediately following OGD (*n* = 7, *p* < 0.0001), increasing during reperfusion, and returning to baseline levels by 12 h. Similar regulation of phosphorylation on residue S727 was observed; phosphorylation was reduced immediately following OGD to 34 ± 7.57% of baseline levels (*n* = 7, *p* < 0.01) returning back to baseline by 6 h of reperfusion.

### 2.2. Inhibition of STAT3 Causes Endothelial Cell Death

**Figure 2:**
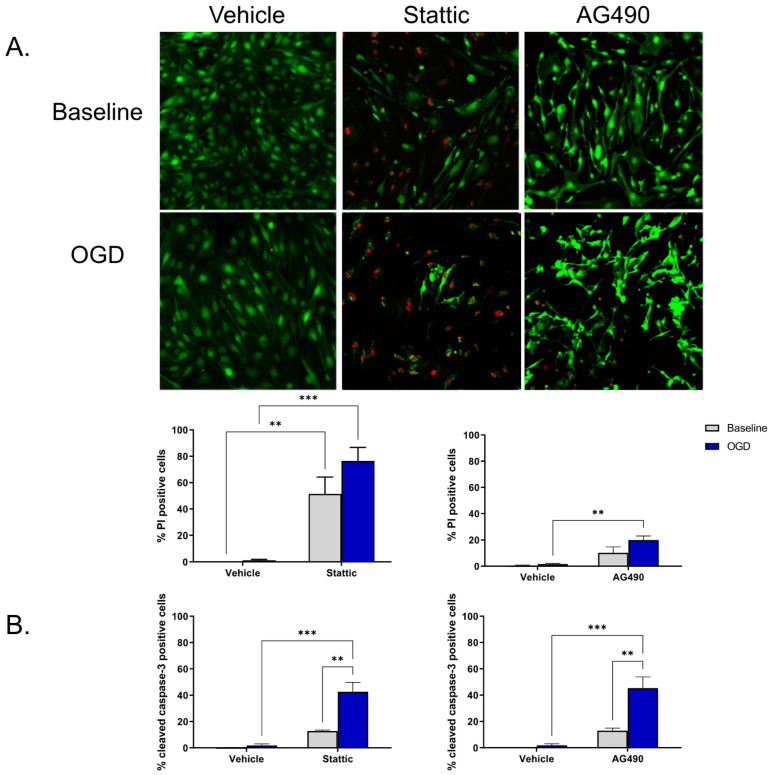
Inhibition of STAT3 causes endothelial cell death. Cells were incubated with STAT3 inhibitors, Stattic (10 μM) or AG490 (1 mM), for 24 h at baseline (no-OGD) or following 8 h OGD. Cell death was assessed by Calcein/PI staining (**A**) and cleaved caspase-3 immunolabeling (**B**). Images show Calcein/PI staining where viable cells appear green, and dead cells red (**A**). Minimal cell death was observed in vehicle-treated ECs both at baseline and following OGD, however cells became more sensitive to OGD following inhibitor treatment. *n* = 3–4; ** *p* < 0.01, *** *p* < 0.001, 2-way ANOVA. Data are presented as mean ± SEM.

To investigate the functional significance of STAT3 activity in ECs, STAT3 activity was pharmacologically reduced using two inhibitors—Stattic and AG490. Stattic inhibits the SH2 domain of STAT3, and AG490 inhibits JAK2 upstream of STAT3. Inhibitors were applied during the 24 h reperfusion period following OGD; cells that had not undergone OGD (baseline) were also treated for the same duration. Cell death was measured using Calcein/propodium iodide (PI) staining as well as by cleaved caspase-3 immunolabeling. We observed that ECs undergo minimal cell death following OGD and subsequent reperfusion (n/s compared to baseline, *n* = 4). Upon inhibition of STAT3, cell death increases both at baseline and following OGD (Figure 2). Application of 1 mM AG490 during reperfusion increases EC death as indicted by the increase in PI positive cells compared to vehicle OGD (19.98 ± 2.95% vs. 1.60 ± 0.60%, respectively, *n* = 4, *p* < 0.01; Figure 2A). PI positive cells were also increased by 10 μM Stattic following OGD compared to vehicle-treated cells, increasing from 1.23% ± 0.66% in vehicle to 76.46% ± 10.21% in Stattic-treated cells (*n* = 3, *p* < 0.001); an increase in cell death of baseline cells was also observed following incubation with Stattic (*n* = 3, *p* < 0.01). To determine whether the cell death observed upon STAT3 inhibition is a consequence of apoptosis, cleaved caspase-3 immunolabeling was used as an additional marker of cell death (Figure 2B). Again, minimal cell death was observed following OGD alone in vehicle-treated cells (1.86 ± 1.17%), which was not significantly different than baseline (0.12 ± 0.12%). On application of either inhibitor, cell death was increased following OGD compared to vehicle OGD (45.40 ± 8.52% vs. 1.86 ± 1.17%, respectively, for AG490; 42.73 ± 6.98 vs. 1.86 ± 1.17, respectively, for Stattic, *n* = 3, *p* < 0.001).

### 2.3. Inhibition of STAT3 Abolishes Endothelial Response to Acetylcholine

**Figure 3:**
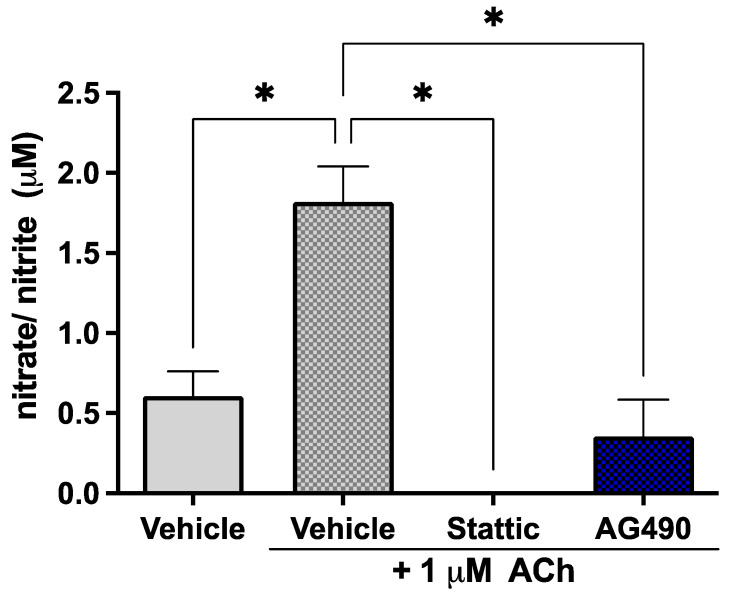
Inhibition of STAT3 abolishes endothelial response to acetylcholine. ECs were treated with Stattic (5 μM) or AG490 (25 μM) for 12 h, following which they were stimulated with 1 μM ACh for 30 min. Medium was collected and analyzed for the metabolites of nitric oxide: nitrate and nitrite. ACh increased nitrate/nitrite release by ECs; treatment with either inhibitor abolished this response to ACh, *n* = 4; *, *p* < 0.05, one-way ANOVA. Data are presented as mean ± SEM.

Since STAT3 phosphorylation is reduced by OGD but little cell death is observed, the effects of sub-lethal STAT3 inhibition were investigated on EC function in order to determine the biological significance of this suppression of STAT3 activity by ischemia. ECs release the vasodilator nitric oxide (NO) in response to acetylcholine (ACh); the response to 1 μM ACh was investigated in our cultures upon sub-lethal inhibition of STAT3 as a measure of endothelial function. The short half-life of NO makes its direct measurement challenging—the levels of its metabolites nitrate/nitrite were therefore measured in culture medium. Cells were treated with 5 μM Stattic or 25 μM AG490 for 12 h, following which they were stimulated with 1 μM ACh; medium was collected for assay after 30 min. Cells were stained with Calcein/PI to ensure that the concentrations used were indeed sub-lethal (Supplementary Figure S1). Inhibition of STAT3 by either AG490 or Stattic abolished the response of ECs to ACh (Figure 3). The observed increase in nitrate/nitrite in the medium in response to 1 μM ACh (1.82 ± 0.22 μM vs. 0.61 ± 0.16 μM in unstimulated cells, *n* = 4, *p* < 0.05) was abolished by AG490 and Stattic, where levels were reduced to that of unstimulated cells (0 ± 0 μM and 0.36 ± 0.23 μM, respectively, *n* = 4, n/s compared to unstimulated). These results were obtained under normoxic conditions; nitrate/nitrite release in response to ACh was also investigated following OGD where there was no nitrate/nitrite production observed under any condition (not shown).

### 2.4. Inhibition of STAT3 Reduces Barrier Integrity of Brain Microvascular Endothelial Cells

**Figure 4:**
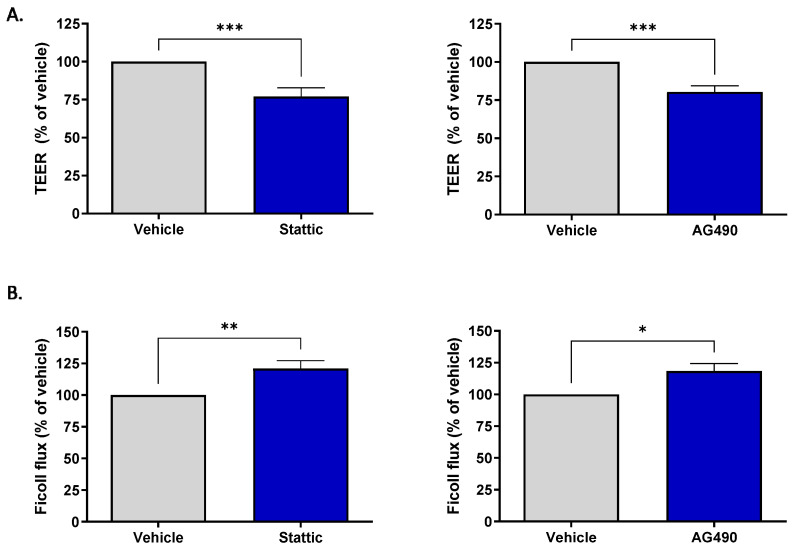
Inhibition of STAT3 reduces barrier integrity of brain microvascular ECs. ECs were incubated with Stattic (1.25 μM) or AG490 (12.5 μM) for 12 h following which barrier integrity was assessed by transendothelial electrical resistance (TEER) and flux of FITC-Ficoll 70 across the monolayer. Treatment with either inhibitor reduced the electrical resistance across the monolayer (**A**) Stattic *n* = 11; AG490 *n* = 10, and increased permeability to Ficoll 70 (**B**), Stattic *n* = 6, AG490 *n* = 6; *, *p* < 0.05; **, *p* < 0.01; ***, *p* < 0.001, t-test. Data are presented as mean ± SEM.

Brain ECs are unique compared to ECs in other vascular beds in their formation of the blood–brain barrier (BBB). This barrier property is the brain’s first line of defense against toxic substances or invading pathogens. The effect of sub-lethal inhibition of STAT3 on barrier function was investigated. Two methods were used to assess barrier function: transendothelial electrical resistance (TEER) and permeability of the endothelial monolayer to passage of fluorescently labeled Ficoll 70. For both assays, cells were plated on transwell inserts and subjected to pharmacological inhibition for 12 h by either 1.25 μM Stattic or 12.5 μM AG490. These concentrations are lower than those used to assess ACh response as the cells were more sensitive to STAT3 inhibition when cultured on transwells; Calcein/PI staining was carried out to ensure that inhibition was sub-lethal (Supplementary Figure S1). Figure 4A shows that treatment with either inhibitor reduced the transendothelial electrical resistance, with Stattic causing a reduction to 77.05 ± 5.71% (*n* = 11, *p* < 0.001) and AG490 to 80.4 ± 3.96% (*n* = 10, *p* < 0.001) of vehicle treated cells. STAT3 inhibition also increased permeability of the endothelial monolayer (Figure 4B). Expressed as a percentage of vehicle, Stattic increased the passage of Ficoll-FITC across the endothelial layer to 120.90 ± 6.25% (*n* = 6, *p* < 0.01) and AG490 to 118.4 ± 5.93% (*n* = 6, *p* < 0.05).

### 2.5. Increased Infarct in Mice with Attenuated Endothelial STAT3

**Figure 5:**
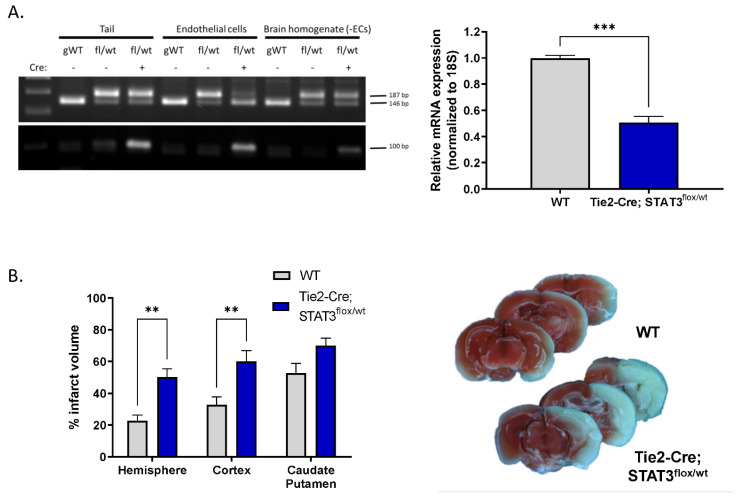
Increased infarct in mice with attenuated endothelial STAT3. (**A**) Cre-mediated attenuation of STAT3 gene in ECs. Left, PCR analysis for WT and floxed STAT3 alleles in DNA isolated from tail, acutely isolated brain ECs, and the remaining brain homogenate minus the extracted ECs from genetically wild-type (gWT), STAT3^flox/wt^ and Tie2-Cre; STAT3^flox/wt^ mice. Amplification of wild-type allele results in a 146 bp fragment; amplification of the floxed allele, containing the loxP sites generates a 187 bp band. Genotyping for the Tie2-Cre allele is also shown; amplification of the Cre transgene results in a 100 bp band. Right, ECs from Tie2-Cre; STAT3^flox/wt^ mice have lower STAT3 mRNA levels compared to ECs from STAT3^flox/wt^ control littermates (WT), measured by TaqMan quantitative real time PCR. *n* = 3; ***, *p* < 0.001, *t*-test. (**B**) Tie2-Cre; STAT3^flox/wt^ and WT mice were subjected to 60 min MCAO, infarct was assessed TTC labeling after 24 h reperfusion, representative brain slices are shown. Mice with conditionally attenuated STAT3 in ECs (Tie2-Cre; STAT3^flox/wt^) displayed larger hemispheric infarct following MCAO compared to WT littermates. *n* = 8 WT, *n* = 6 Tie2-Cre; STAT3^flox/wt^; **, *p* < 0.01, 2-way ANOVA. Data are presented as mean ± SEM.

To determine whether the endothelial dysfunction we observe in vitro upon pharmacological inhibition of STAT3 has functional relevance in vivo, we investigated the effect of reduced endothelial STAT3 on outcome after experimental stroke. MCAO was performed on mice with reduced endothelial STAT3 (STAT3^flox/wt^) and compared with WT littermates. Mice were generated by crossing mice in which STAT3 exons 18–20 are flanked by loxP sequences with Tie2-Cre mice, resulting in the removal of the Src homology 2 domain of STAT3 required for protein function, specifically in ECs [23,24]. Genotype was verified by PCR of tail DNA (Figure 5A). The reduction in STAT3 levels in ECs isolated from these mice was verified by real-time quantitative PCR (Figure 5A) demonstrating that relative STAT3 transcript is reduced from 1 (0.81–1.23) in WT mice to 0.5 (0.41–0.62) in Tie2-Cre; STAT3^flox/wt^ mice (*n* = 3, *p* < 0.001). Mice were subjected to 60 min MCAO and infarct size assessed following 24 h of reperfusion by 2,3,5-triphenyltetrazolium chloride (TTC) labeling. Tie2-Cre; STAT3^flox/wt^ mice sustained larger infarcts following MCAO (Figure 5B) compared to WT. Hemispheric infarct was increased over 2-fold from 22.64 ± 3.74% in WT to 50.28 ± 5.07% in Tie2-Cre; STAT3^flox/wt^ (*n* = 6–8, *p* < 0.01). This increase appears largely dirven by increased infarct in the cortex in Tie2-Cre; STAT3^flox/wt^ mice (32.76 ± 5.06% WT vs. 60.00 ± 6.90% in Tie2-Cre; STAT3^flox/wt^; *n* = 6–8, *p* < 0.01), with no significant change observed in the caudate putamen.

## 3. Discussion

Global pharmacological inhibition of STAT3 exacerbates ischemic brain damage following stroke [9], however it is not clear which cell type(s) mediate this effect. The role of endothelial STAT3 signaling in cerebral ischemia/reperfusion has not been fully elucidated. We report that STAT3 is constitutively active in primary mouse cerebrovascular ECs in vitro, and that its phosphorylation is decreased by ischemia, recovering during reperfusion. Functionally, we demonstrate that inhibiting STAT3 in ECs is sufficient to increase endothelial barrier permeability and decrease response to ACh, mimicking the effect of ischemia. Finally, we show that mice with attenuated endothelial STAT3 sustain larger infarcts compared to WT controls 24 h following MCAO, indicating that endothelial STAT3 contributes to the tissue protection observed with pharmacological inhibition of STAT3.

As the luminal-most cell of the blood–brain barrier, ECs are the brain’s first line of defense against blood-borne toxic agents. We report here that cerebral endothelial cells are remarkably resistant to cell death following ischemia—following 8 h of OGD minimal cell death was observed. This is in contrast to other constituent brain cell types which are more sensitive to cell death following ischemia: 6 h of OGD induces 50% cell death in astrocytes and as little as 2 h of OGD results in 40% decreased survival of cultured neurons [25,26]. We show that the up-regulation of activated STAT3 during reperfusion is an important contributor to the resistance of ECs to death following OGD, as inhibiting this increase in STAT3 activation causes significant cell death and exacerbates OGD-induced cell death, indicating that the duration of STAT3 inhibition may play a role in determining the extent of cell death. A limitation of both methods employed to assess cell death (Calcein/PI and cleaved caspase-3 labeling) is that they do not account for cell detachment upon death, therefore potentially underestimating the extent of cell death elicited by STAT3 inhibition.

Interestingly, our data demonstrates regulation of STAT3 phosphorylation on serine residue 727 (S727), in addition to tyrosine 705 (Y705). Canonically, STAT3 is activated by tyrosine phosphorylation downstream of a variety of cytokines and growth factors. This convergence of multiple signaling pathways on STAT3 results in many distinct, sometimes contradictory, functions of STAT3 signaling. Phosphorylation on S727 occurs by members of the mitogen-activated protein kinases (MAPK) and c-Jun-N-terminal kinase families though can also be phosphorylated by cytokine receptors. The role serine phosphorylation plays in STAT3 activity remains controversial; it is required for optimal transcription of a subset of genes, although it can also inhibit further phosphorylation of the tyrosine residue [27,28,29,30,31]. Serine phosphorylation has been implicated in mitochondrial import of STAT3 [27,32]. In mitochondria, STAT3 functions include supporting electron transport chain activity required for ATP production, opening of the permeability pore, and transcription of mitochondrial DNA encoded genes [33]. The original description of mitochondrial activity of STAT3 was made following ischemia/reperfusion injury in cardiomyocytes [34]. The role of mitochondrial STAT3 in ECs following ischemia has not been explored; however, compromised mitochondrial function in cerebrovascular ECs contributes to increased BBB permeability [35]. It is therefore possible that the serine regulation we observe following ischemia may be linked to increased endothelial barrier permeability via its non-canonical role in the mitochondria.

We show that STAT3 is involved in the maintenance of barrier integrity, with sub-lethal inhibition of STAT3 resulting in increased permeability across the endothelial monolayer and decreased electrical resistance. These are both prominent characteristics of endothelial- and BBB dysfunction, implicated in ischemic pathology [18,21]. In ischemic stroke, BBB disruption can lead to increased inflammatory responses due to infiltrating leukocytes and edema, and is associated with poor prognosis [36]. Temporally, BBB leakage occurs prior to infarct development [37], with experimental manipulations aimed at decreasing BBB dysfunction leading to improved outcome [37,38]. Our results suggest that interventions aimed at activating endothelial STAT3 in the acute stages of ischemic stroke may be tissue protective, in part via maintenance of barrier integrity. Interestingly, in the recovery phase after stroke, endothelial STAT3 plays a role in promoting angiogenesis [12]. Endothelial STAT3 can have pro-angiogenic functions in paradigms other than stroke, where stimulation of ECs by vascular endothelial growth factor (VEGF) leads to STAT3-mediated angiogenesis and associated vascular permeability via canonical STAT3 signaling [39,40,41]. However, endothelial STAT3-mediated angiogenesis (observed 28 days post MCAO), a key feature of long-term stroke recovery, is independent of VEGF signaling [12]. As discussed, STAT3 is a pleiotropic factor with strongly context-specific activity by virtue of activation by a variety of factors and subsequent regulation of many different genes. Following ischemia, endothelial STAT3 may have multiple, opposing, functions regarding the BBB, transducing the effects of multiple factors, with temporally distinct profiles, perhaps mediated by a combination of canonical and non-canonical signaling.

Regulation of vascular tone and blood flow is an important function of ECs, brought about by endothelial release of nitric oxide. Impairment of the NO generating enzyme, endothelial nitric oxide synthase (eNOS), by ischemic stroke is implicated in the pathogenesis of endothelial dysfunction, neuronal injury and development of subsequent infarct [42]. We show that STAT3 is involved in the NO response to ACh, as STAT3 inhibition abolished the increase in nitrate/nitrite in response to ACh in brain EC cultures. Regulation of NOS by STAT3 suggests that endothelial STAT3 contributes to protection from ischemia by at least two mechanisms, both of which have been linked to NO: (1) blood flow regulation, and (2) BBB integrity. Our findings add the role of STAT3 in NO signaling in brain ECs to what is known in the aorta where AG490 decreases both eNOS phosphorylation and NOS activity [43]. Others however have reported that STAT3 can inhibit eNOS protein expression [44,45,46], raising the possibility of a dual role for STAT3 in regulating eNOS expression and activity.

We demonstrate increased infarct in mice with attenuated endothelial STAT3 24 h after MCAO. A previous study demonstrated a protective role of STAT3 in stroke with pharmacological inhibition leading to increased infarct [9]; however, such a study does not inform about which cell type(s) mediate this effect. In the long-term, endothelial STAT3 supports angiogenesis and recovery, with endothelial knock-out resulting in reduced angiogenesis and increased infarct 28 days post-MCAO [12]. We show that a reduction, in contrast to full-knock-out, of endothelial STAT3 levels is sufficient to double infarct size in the acute phase, 1 day following MCAO, demonstrating tissue-protective effects of STAT3 independent of post-stroke angiogenesis. Endothelial dysfunction contributes to the development of ischemic lesions following cerebral ischemia [36]. We show that inhibition of STAT3, as occurs during ischemia, is sufficient to induce endothelial dysfunction measured by response to ACh and barrier integrity. This endothelial dysfunction may contribute to the pathogenesis of surrounding brain tissue following MCAO, a phenomenon exacerbated in our mouse model of attenuated endothelial STAT3. Our mouse model is that of chronically reduced STAT3, which may render ECs dysfunctional prior to ischemic insult, and may have further physiological relevance since a reduction in brain STAT3 protein levels is observed in aging, a risk factor for stroke [47].

This present study identifies STAT3 as a factor important in brain microvascular EC function and survival. We demonstrate that sub-lethal inhibition of STAT3 is sufficient to cause endothelial dysfunction, and that endothelial specific attenuation of STAT3 leads to increased infarct following MCAO. Together our results suggest that STAT3 helps maintain the integrity of the blood–brain barrier and that its down-regulation during ischemic events contributes to pathological brain injury, identifying endothelial STAT3 as a potential therapeutic target in stroke.

## 4. Materials and Methods

Studies were performed according to the National Institutes of Health Guidelines for the use and care of laboratory animals, and protocols were approved by the OHSU Institutional Animal Care and Use Committee. Reporting of results conforms to ARRIVE 2.0 (Animal Research: Reporting on In Vivo Experiments) guidelines [48].

### 4.1. Primary Cerebral Microvessel Endothelial Cell Culture

Primary mouse cerebral microvascular ECs were isolated and cultured from 10-week-old male C57BL/6 mice (Charles River Laboratory, Worcester, MA, USA), as previously described and characterized [49,50]. Briefly, cerebral cortices harvested from 5 mice, lacking cerebella, large vessels and leptomeninges by dissection and rolling on filter paper, were digested with 1 mg/mL collagenase CLS2 (Worthington) and 0.01 mg/mL DNase I (Sigma) in Dulbecco’s Modified Eagle Medium (DMEM) for 60 min at 37 °C with agitation. Cells were pelleted by centrifugation for 10 min at 1000 rpm. Myelin was removed by resuspending the pellet in DMEM containing 20% bovine serum albumin (BSA) and subsequent centrifugation for 10 min at 1000 rpm, the microvessels in the remaining pellet were further digested with 1 mg/mL collagenase/dispase (Roche) and 0.01 mg/mL DNase I in DMEM for 30 min at 37 °C and subsequently separated on a 33% Percoll gradient (GE Healthcare) by centrifugation at 1000 rpm for 20 min at 4 °C. Microvessels were washed once with DMEM, pelleted and plated in three collagen IV (Sigma)-coated T25 cell culture flasks in high glucose (4.5 g/L) DMEM supplemented with 20% fetal bovine serum (FBS), 50 µg/mL gentamycin, 2 mM glutamine, 100 µg/mL heparin, 100 µg/mL ECGS, and endothelial mitogen (Biomedical Technologies Inc), also containing 4 µg/mL puromycin (Sigma) for the first 36 h. Once confluent, cells were detached (0.05% trypsin-EDTA, Sigma-Aldrich) and plated on collagen IV-coated multiwell plates for experiments at a density of 2 × 10^4^ for 48 well plates (cell death and nitrate/nitrite assays) and 6 × 10^4^ for 12 well plates (Western blotting) and transwells; cells were grown to confluence before commencing experiments.

### 4.2. Oxygen-Glucose Deprivation (OGD)

ECs were subjected to OGD for 8 h at 37 °C in an anaerobic chamber (Coy Laboratory Products, Grass Lake, MI, USA) filled with an anoxic gas mixture (5% CO_2_, 5% H_2_, and 90% N_2_). Oxygen concentration was maintained at 0 parts per million using a palladium catalyst. Anoxic conditions were monitored with an oxygen monitor (Oxygen-Hydrogen Gas Analyzer; Coy Laboratory Products) inside the chamber. To initiate OGD, the culture medium was removed, and cells were rinsed 3 times with DPBS and replaced with glucose-free DMEM before placement in the anaerobic chamber. OGD was terminated by removing cells from the chamber and replacing medium with normal culture medium (defined above).

### 4.3. Western Blotting

Cells were lysed in buffer containing HEPES (0.01 mol/L), potassium chloride (0.01 mol/L), ethylenediaminetetraacetic acid (0.1 mmol/L), ethylene glycol tetraacetic acid (0.1 mmol/L), phenylmethanesulfonylfluoride (0.5 mmol/L), dithiothreitol (1 mmol/L), cOmplete Mini-EDTA free Protease Inhibitor Cocktail (1 tablet per 10 mL; Roche Diagnostics, Indianapolis, IN, USA), and 10 μL/mL each of phosphatase inhibitor solution 2 and phosphatase inhibitor solution 3 (Sigma-Aldrich, St Louis, MO, USA) and 0.5% NP-40. Lysates were centrifuged at 12,000 g for 10 min at 4 °C and supernatant collected. Protein samples (40 μg) were separated by gel electrophoresis on a 4–12% Bis-Tris Gel (Life Technologies) and transferred onto polyvinylidene difluoride membranes. Blots were blocked in 5% dry milk, and incubated at 4 °C overnight with primary antibody; phospho-Stat3 (Tyr705: 1:1000; #9145), phospho-Stat3 (Ser727; 1:1000; #9134) and Stat3 (1:1000; #5880; all Cell Signaling Technology Inc.). The signal was visualized using a horseradish peroxidase-linked rabbit secondary antibody (1:1000; GE Healthcare Life Sciences, Piscataway, NJ, USA) followed by detection using SuperSignal West Dura Chemiluminescent Substrate (Thermo Fisher Scientific, Waltham, MA, USA) with an Alpha Innotech FluorChem FC2 Imager (R&D Systems, Minneapolis, MN, USA). Blots were stripped using Restore Western Blot Stripping Buffer (Thermo Fisher Scientific) and re-probed for GAPDH (1:2000; #A5-15738; Thermo Fisher Scientific) followed by horseradish peroxidase- linked mouse secondary antibody (1:1000; GE Healthcare Life Sciences) and reimaged. Densitometry was quantified using the AlphaView software (Protein Simple, Santa Clara, CA, USA); phospho-Stat3 was normalized relative to STAT3.

### 4.4. Cell Death Assays

Cell viability was assessed by staining cells with fluorescent markers for live (Calcein-AM, Invitrogen, Carlsbad, CA, USA) and dead (Propidium Iodide (PI), Sigma-Aldrich) cells. Briefly, Calcein-AM solution (reconstituted in dimethyl sulfoxide) was added to confluent ECs in a 48-well plate, to a final concentration of 1 μg/mL, and incubated at 37 °C. After 15 min PI was added to each well, to a final concentration of 3.75 μg/mL and incubated for a further 15 min. Cells were examined under an inverted fluorescence microscope (Nikon TE200, Nikon, Tokyo, Japan); live cells appear green and dead cells red. Cell death was calculated from the ratio of PI-positive cells to the sum of PI-positive and Calcein-AM–positive cells (if a cell contained both green and red labeling, it was counted as red). Cell death was also assessed by immunolabeling ECs for cleaved caspase-3 (below). Four images were acquired of every culture well (one image/quadrant), and conditions were run in duplicate (i.e., 2 wells/condition) for every experiment.

### 4.5. Immunocytochemistry

Cells were fixed in culture plates using 4% paraformaldehyde and blocked for 1 h at room temperature in 5% normal donkey serum + 1% BSA + 0.1% TritonX-100 solution. Cleaved caspase-3 antibody (1:250, Cell Signaling Technology Inc., Danvers, MA, USA) diluted in blocking buffer, was applied and incubated overnight at 4 °C. Cells were washed with PBS + 0.1% Tween-20, and secondary antibody and Hoechst (1:1000; Life Technologies) were applied in blocking buffer for 2 h at room temperature. Cells were washed and examined under an inverted fluorescence microscope (Nikon TE200, Nikon, Tokyo, Japan).

### 4.6. Transendothelial Electrical Resistance (TEER)

Cells were grown to confluence in culture medium on collagen IV-coated transwell permeable supports (0.4 μm polyester membrane, 12 mm insert; Costar, NY, USA), culture medium was replaced with EGM-2 MV (minus the VEGF supplement; Lonza, Basel, Switzerland) for 72 h. TEER was measured before and after inhibitor treatment using a Microvolt-ohmmeter and concentric-ring electrode system (EndOhm, World Precision Instruments; Sarasota, FL, USA). After the transwell was placed in the chamber, readings were allowed to stabilize for 10 s before recording. The value was multiplied by the surface area of the membrane support to obtain TEER in Ω/cm^2^. Once approximately 20–25 Ω/cm^2^ was reached, Stattic/AG490 were added for 12 h. This reading is in accordance with other studies for brain endothelial cells cultured in the absence of astrocytes/pericytes [51]. Since the resistance of each transwell was not identical before treatment, results were calculated by expressing the post-inhibitor resistance as a percentage of its pre-inhibitor value, normalized to vehicle.

### 4.7. Permeability Assay

Following TEER measurement, the same cells were used to study permeability across the endothelial monolayer. Medium in upper and lower compartments of the transwell was changed to phenol-red free DMEM. FITC-Ficoll 70 (TdB Consultancy, Uppsala, Sweden) was added to a final concentration of 9.4 mg/mL in the upper chamber of the transwell support. After 45 min, samples were taken from the bottom compartment and measured on a fluorescent plate reader (Victor 3, Perkin Elmer, Waltham, MA, USA) using excitation and emission wavelengths of 405 nM and 535 nM, respectively, to measure extravasated FITC-Ficoll from the top to bottom chamber across the endothelial monolayer.

### 4.8. Nitrate/Nitrite Assay

Nitrate/Nitrite Fluorometric Assay Kit (Cayman Chemical Company, Ann Arbor, MI, USA) was carried out according to manufacturer’s instructions. Cells were cultured in 48 well plates. Just prior to experiment, culture medium was replaced with phenol red free DMEM, and cells were stimulated with ACh (1 μM) for 30 min. STAT3 pharmacological inhibitors were added 24 h prior to experiment and were also present in the phenol red free DMEM used for the assay.

### 4.9. Endothelial-Specific STAT3 Knock-Out Mice

STAT3 floxed mice have exons 18–20 flanked by loxP sites, as previously described [23] were provided by Dr. X. Y. Fu at The National University of Singapore, and have been backcrossed into the C57Bl/6 background for at least 5 generations. Primer sequences for genotyping by PCR are: fwd 5′-TTG ACC TGT GCT CCT ACA AAA A-3′ and rev 5′-CCC TAG ATT AGG CCA GCA CA-3′. Wild type and floxed STAT3 alleles yield 146 and 187 bp products, respectively. The Tie2-Cre (B6.Cg-Tg(Tek-cre)12Flv/J) mice were provided by Dr. W Fleming, Oregon Health and Science University [24], and have been backcrossed into the C57Bl/6 background for at least 14 generations. The primers used for genotyping are: fwd 5′-GCG GTC TGG CAG TAA AAA CTA TC-3′ and rev 5′-GTG AAA CAG CAT TGC TGT CAC TT-3′, producing a band of 100 bp if the Cre transgene is present. The mice used for MCAO studies were generated by breeding STAT3^flox/flox^ mice with Tie2-Cre mice. The resulting mice are heterozygous for the STAT3 floxed allele and either Cre positive (Tie2-Cre; STAT3^flox/wt^), or Cre negative, referred to as WT. Animals were housed in a temperature- and humidity-controlled facility under a 12 h dark/light cycle, and fed standard mouse chow and water ad libitum.

### 4.10. Acute Isolation of Endothelial Cells

ECs were acutely isolated from mouse cortices. Briefly, tissue was digested using collagenase CLS2 (Worthington Biochemical Corporation, Lakewood, NJ, USA) for 1 h with agitation, triturated and centrifuged at 1000 rpm for 10 min, cells were re-suspended in PBS containing 0.1% BSA and incubated with Dynabeads (M-450 sheep anti-rat IgG; Life Technologies) conjugated to rat anti-mouse CD102 antibody (BD Pharmingen, NJ, USA) at room temperature for 40 min to allow binding of the Dynabeads/antibody to ECs. ECs were then harvested using a magnetic separator (Dynal, Life Technologies).

### 4.11. DNA Isolation

CD102 Dynabeads-bound cells (ECs) were re-suspended in PBS. Unbound cells from the brain homogenate (flow through in magnetic separator) were pelleted by centrifugation at 1000 rpm for 10 min, and re-suspended in PBS. DNA was isolated using DNeasy Blood & Tissue Kit (QIAGEN, Valencia, CA, USA), according to manufacturer’s instructions.

### 4.12. TaqMan Real-Time Quantitative Polymerase Chain Reaction

RNA was isolated using RNAqueous-Micro kit (Ambion, Grand Island, NY, USA) and reverse transcribed using the High Capacity cDNA Reverse Transcription Kit (ABI, Grand Island, NY, USA). The resulting cDNA was amplified using TaqMan Universal polymerase chain reaction (PCR) amplification with STAT3 TaqMan Gene Expression Assay (Mm0129775-m1; ABI) in the ABI Prism 7000 sequence detection system (Applied Biosystems, Grand Island, NY, USA). Quantitative PCR was performed in a 96-well plate, with each sample run in triplicate. PCR was also run on controls in which the template had not been added to determine DNA contamination and primer–dimer formation. RNA that had not been reverse transcribed was also included to discount genomic DNA amplification. 18S was measured as an internal control using the 18S rRNA control kit-FAM-TAMRA (Eurogentec, Liege, Belgium). Data was analyzed using the comparative CT method.

### 4.13. Middle Cerebral Artery Occlusion (MCAO)

All mice were subjected to transient focal cerebral ischemia by use of the intraluminal MCAO technique [52]. Eight- to twelve-week-old (20–25 g) male Tie2-Cre; STAT3^flox/wt^ and WT littermate mice were anesthetized with isoflurane and kept warm using water pads. Induction of anesthesia was achieved using 5% isoflurane mixed with 0.1 L/min oxygen and 0.5 L/min air, decreasing to 2% isoflurane for set-up of mouse on the surgical station and 1.5% for surgery. A transcranial laser-Doppler probe (MBF3D, Moor Instruments, Oxford, UK) was secured on the right temporal side of the head to monitor cerebrocortical perfusion and verify vascular occlusion and reperfusion. A silicone-coated 6-0 nylon monofilament was advanced into the right internal carotid artery via the external carotid artery until laser-Doppler signal dropped to below 30% of baseline. Mice were maintained anesthetized with 1% isoflurane on the surgical station for 60 min of occlusion. At the end of occlusion, the filament was withdrawn for reperfusion and the mice were allowed to recover. All surgeries were carried out by the same surgeon.

Infarct size was measured 24 h following MCAO in 2 mm thick coronal sections using 2,3,5-triphenyltetrazolium chloride (TTC) staining. Sections were incubated for 15 min in 1.2% TTC (Sigma-Aldrich) in saline at 37 °C and fixed in formalin for 24 h. Sections (5 per animal) were imaged, infarcted (unstained) and viable tissue (stained) areas were measured using MCID software (InterFocus, Linton, UK) and integrated across all 5 sections. To discount the influence of edema, infarct size was calculated indirectly by subtracting the uninjured area in the ipsilateral hemisphere from the contralateral hemisphere, then expressing infarct volume as a percentage of the contralateral hemisphere.

### 4.14. Statistical Analysis

Differences between two groups were evaluated with a *t*-test; those for many groups were evaluated by one-way or two-way ANOVA, as appropriate, with Tukey’s multiple comparisons post hoc test using GraphPad Prismm version 9.4.0. The criterion for statistical significance was set at *p <* 0.05. All values are reported as mean +/− S.E.M.

## Data Availability

The data presented in this study are available on request from the corresponding author.

## Supplementary Materials

The following supporting information can be downloaded at: https://www.mdpi.com/article/10.3390/ijms232012167/s1, Figure S1: Calcein/PI labeling of ECs incubated for 12 h with 5 µM Stattic and 25 µM AG490 (12-well plate) or 1.25 µM Stattic and 12.5 µM AG490 (transwell) demonstrating that these concentrations are sub-lethal. High dose DMSO was used as a positive (cell death) control. Scale bar, 100 µm.

## References

[1] Dziennis S., Alkayed N.J. (2008). Role of signal transducer and activator of transcription 3 in neuronal survival and regeneration. Rev. Neurosci..

[2] Suzuki S., Tanaka K., Nogawa S., Dembo T., Kosakai A., Fukuuchi Y. (2001). Phosphorylation of signal transducer and activator of transcription-3 (Stat3) after focal cerebral ischemia in rats. Exp. Neurol..

[3] Amantea D., Tassorelli C., Russo R., Petrelli F., Morrone A.L., Bagetta G., Corasaniti M.T. (2011). Neuroprotection by leptin in a rat model of permanent cerebral ischemia: Effects on STAT3 phosphorylation in discrete cells of the brain. Cell Death Dis..

[4] Qiu J., Yan Z., Tao K., Li Y., Li Y., Li J., Dong Y., Feng D., Chen H. (2016). Sinomenine activates astrocytic dopamine D2 receptors and alleviates neuroinflammatory injury via the CRYAB/STAT3 pathway after ischemic stroke in mice. J. Neuroinflamm..

[5] Levy D.E., Darnell J.E. (2002). Stats: Transcriptional control and biological impact. Nat. Rev. Mol Cell Biol.

[6] Shen Y., Devgan G., Darnell J.E., Bromberg J.F. (2001). Constitutively activated Stat3 protects fibroblasts from serum withdrawal and UV-induced apoptosis and antagonizes the proapoptotic effects of activated Stat1. Proc. Natl. Acad. Sci. USA.

[7] Lapp D.W., Zhang S.S., Barnstable C.J. (2014). Stat3 mediates LIF-induced protection of astrocytes against toxic ROS by upregulating the UPC2 mRNA pool. Glia.

[8] Benito C., Davis C.M., Gomez-Sanchez J.A., Turmaine M., Meijer D., Poli V., Mirsky R., Jessen K.R. (2017). STAT3 Controls the Long-Term Survival and Phenotype of Repair Schwann Cells during Nerve Regeneration. J. Neurosci..

[9] Dziennis S., Jia T., Rønnekleiv O.K., Hurn P.D., Alkayed N.J. (2007). Role of Signal Transducer and Activator of Transcription-3 in Estradiol-Mediated Neuroprotection. J. Neurosci..

[10] Jung J.E., Kim G.S., Narasimhan P., Song Y.S., Chan P.H. (2009). Regulation of Mn-superoxide dismutase activity and neuroprotection by STAT3 in mice after cerebral ischemia. J. Neurosci..

[11] Sehara Y., Sawicka K., Hwang J.Y., Latuszek-Barrantes A., Etgen A.M., Zukin R.S. (2013). Survivin Is a transcriptional target of STAT3 critical to estradiol neuroprotection in global ischemia. J. Neurosci..

[12] Hoffmann C.J., Harms U., Rex A., Szulzewsky F., Wolf S.A., Grittner U., Lättig-Tünnemann G., Sendtner M., Kettenmann H., Dirnagl U. (2015). Vascular signal transducer and activator of transcription-3 promotes angiogenesis and neuroplasticity long-term after stroke. Circulation.

[13] Li L., Li H., Li M. (2015). Curcumin protects against cerebral ischemia-reperfusion injury by activating JAK2/STAT3 signaling pathway in rats. Int. J. Clin. Exp. Med..

[14] Sharma S., Yang B., Xi X., Grotta J.C., Aronowski J., Savitz S.I. (2011). IL-10 directly protects cortical neurons by activating PI-3 kinase and STAT-3 pathways. Brain Res..

[15] Chen S., Dong Z., Cheng M., Zhao Y., Wang M., Sai N., Wang X., Liu H., Huang G., Zhang X. (2017). Homocysteine exaggerates microglia activation and neuroinflammation through microglia localized STAT3 overactivation following ischemic stroke. J. Neuroinflamm..

[16] Ding Y., Qian J., Li H., Shen H., Li X., Kong Y., Xu Z., Chen G. (2019). Effects of SC99 on cerebral ischemia-perfusion injury in rats: Selective modulation of microglia polarization to M2 phenotype via inhibiting JAK2-STAT3 pathway. Neurosci. Res..

[17] Zhang Y., Liu J., Yang B., Zheng Y., Yao M., Sun M., Xu L., Lin C., Chang D., Tian F. (2018). Ginkgo biloba Extract Inhibits Astrocytic Lipocalin-2 Expression and Alleviates Neuroinflammatory Injury via the JAK2/STAT3 Pathway After Ischemic Brain Stroke. Front. Pharmacol..

[18] Deli M.A., Abrahám C.S., Kataoka Y., Niwa M. (2005). Permeability studies on in vitro blood-brain barrier models: Physiology, pathology, and pharmacology. Cell Mol. Neurobiol..

[19] del Zoppo G.J., Milner R. (2006). Integrin-matrix interactions in the cerebral microvasculature. Arterioscler. Thromb. Vasc. Biol..

[20] Abbott N.J., Patabendige A.A., Dolman D.E., Yusof S.R., Begley D.J. (2010). Structure and function of the blood-brain barrier. Neurobiol. Dis..

[21] Petito C.K. (1979). Early and late mechanisms of increased vascular permeability following experimental cerebral infarction. J. Neuropathol. Exp. Neurol..

[22] Sandoval K.E., Witt K.A. (2008). Blood-brain barrier tight junction permeability and ischemic stroke. Neurobiol. Dis..

[23] Welte T., Zhang S.S., Wang T., Zhang Z., Hesslein D.G., Yin Z., Kano A., Iwamoto Y., Li E., Craft J.E. (2003). STAT3 deletion during hematopoiesis causes Crohn’s disease-like pathogenesis and lethality: A critical role of STAT3 in innate immunity. Proc Natl. Acad. Sci. USA.

[24] Bailey A.S., Willenbring H., Jiang S., Anderson D.A., Schroeder D.A., Wong M.H., Grompe M., Fleming W.H. (2006). Myeloid lineage progenitors give rise to vascular endothelium. Proc. Natl. Acad. Sci. USA.

[25] Liu M., Alkayed N.J. (2005). Hypoxic preconditioning and tolerance via hypoxia inducible factor (HIF) 1alpha-linked induction of P450 2C11 epoxygenase in astrocytes. J. Cereb. Blood Flow Metab..

[26] Mao P., Ardeshiri A., Jacks R., Yang S., Hurn P.D., Alkayed N.J. (2007). Mitochondrial mechanism of neuroprotection by CART. Eur. J. Neurosci..

[27] Avalle L., Poli V. (2018). Nucleus, Mitochondrion, or Reticulum? STAT3 à La Carte. Int. J. Mol. Sci..

[28] Decker T., Kovarik P. (2000). Serine phosphorylation of STATs. Oncogene.

[29] Yang J., Kunimoto H., Katayama B., Zhao H., Shiromizu T., Wang L., Ozawa T., Tomonaga T., Tsuruta D., Nakajima K. (2020). Phospho-Ser727 triggers a multistep inactivation of STAT3 by rapid dissociation of pY705-SH2 through C-terminal tail modulation. Int. Immunol..

[30] Qin H.R., Kim H.J., Kim J.Y., Hurt E.M., Klarmann G.J., Kawasaki B.T., Duhagon Serrat M.A., Farrar W.L. (2008). Activation of signal transducer and activator of transcription 3 through a phosphomimetic serine 727 promotes prostate tumorigenesis independent of tyrosine 705 phosphorylation. Cancer Res..

[31] Shi X., Zhang H., Paddon H., Lee G., Cao X., Pelech S. (2006). Phosphorylation of STAT3 serine-727 by cyclin-dependent kinase 1 is critical for nocodazole-induced mitotic arrest. Biochemistry.

[32] Tammineni P., Anugula C., Mohammed F., Anjaneyulu M., Larner A.C., Sepuri N.B. (2013). The import of the transcription factor STAT3 into mitochondria depends on GRIM-19, a component of the electron transport chain. J. Biol. Chem..

[33] Garama D.J., White C.L., Balic J.J., Gough D.J. (2016). Mitochondrial STAT3: Powering up a potent factor. Cytokine.

[34] Wegrzyn J., Potla R., Chwae Y.J., Sepuri N.B., Zhang Q., Koeck T., Derecka M., Szczepanek K., Szelag M., Gornicka A. (2009). Function of mitochondrial Stat3 in cellular respiration. Science.

[35] Doll D.N., Hu H., Sun J., Lewis S.E., Simpkins J.W., Ren X. (2015). Mitochondrial crisis in cerebrovascular endothelial cells opens the blood-brain barrier. Stroke.

[36] Jiang X., Andjelkovic A.V., Zhu L., Yang T., Bennett M.V.L., Chen J., Keep R.F., Shi Y. (2018). Blood-brain barrier dysfunction and recovery after ischemic stroke. Prog. Neurobiol..

[37] Shi Y., Zhang L., Pu H., Mao L., Hu X., Jiang X., Xu N., Stetler R.A., Zhang F., Liu X. (2016). Rapid endothelial cytoskeletal reorganization enables early blood-brain barrier disruption and long-term ischaemic reperfusion brain injury. Nat. Commun..

[38] Shi Y., Jiang X., Zhang L., Pu H., Hu X., Zhang W., Cai W., Gao Y., Leak R.K., Keep R.F. (2017). Endothelium-targeted overexpression of heat shock protein 27 ameliorates blood-brain barrier disruption after ischemic brain injury. Proc. Natl. Acad. Sci. USA.

[39] Wei D., Le X., Zheng L., Wang L., Frey J.A., Gao A.C., Peng Z., Huang S., Xiong H.Q., Abbruzzese J.L. (2003). Stat3 activation regulates the expression of vascular endothelial growth factor and human pancreatic cancer angiogenesis and metastasis. Oncogene.

[40] Chen S.H., Murphy D.A., Lassoued W., Thurston G., Feldman M.D., Lee W.M. (2008). Activated STAT3 is a mediator and biomarker of VEGF endothelial activation. Cancer Biol. Ther..

[41] Terpolilli N.A., Moskowitz M.A., Plesnila N. (2012). Nitric oxide: Considerations for the treatment of ischemic stroke. J. Cereb. Blood Flow Metab..

[42] Wang L., Astone M., Alam S.K., Zhu Z., Pei W., Frank D.A., Burgess S.M., Hoeppner L.H. (2021). Suppressing STAT3 activity protects the endothelial barrier from VEGF-mediated vascular permeability. Dis. Model. Mech..

[43] Zecchin H.G., Priviero F.B., Souza C.T., Zecchin K.G., Prada P.O., Carvalheira J.B., Velloso L.A., Antunes E., Saad M.J. (2007). Defective insulin and acetylcholine induction of endothelial cell-nitric oxide synthase through insulin receptor substrate/Akt signaling pathway in aorta of obese rats. Diabetes.

[44] McCormick M.E., Goel R., Fulton D., Oess S., Newman D., Tzima E. (2011). Platelet-endothelial cell adhesion molecule-1 regulates endothelial NO synthase activity and localization through signal transducers and activators of transcription 3-dependent NOSTRIN expression. Arterioscler. Thromb. Vasc. Biol..

[45] Saura M., Zaragoza C., Bao C., Herranz B., Rodriguez-Puyol M., Lowenstein C.J. (2006). Stat3 mediates interleukin-6 [correction of interelukin-6] inhibition of human endothelial nitric-oxide synthase expression. J. Biol. Chem..

[46] Sud N., Black S.M. (2009). Endothelin-1 impairs nitric oxide signaling in endothelial cells through a protein kinase Cdelta-dependent activation of STAT3 and decreased endothelial nitric oxide synthase expression. DNA Cell Biol..

[47] De-Fraja C., Conti L., Govoni S., Battaini F., Cattaneo E. (2000). STAT signalling in the mature and aging brain. Int. J. Dev. Neurosci..

[48] Percie du Sert N., Hurst V., Ahluwalia A., Alam S., Avey M.T., Baker M., Browne W.J., Clark A., Cuthill I.C., Dirnagl U. (2020). The ARRIVE guidelines 2.0: Updated guidelines for reporting animal research. J. Cereb. Blood Flow Metab..

[49] Gupta N.C., Davis C.M., Nelson J.W., Young J.M., Alkayed N.J. (2012). Soluble epoxide hydrolase: Sex differences and role in endothelial cell survival. Arterioscler. Thromb. Vasc. Biol..

[50] Davis C.M., Ammi A.Y., Alkayed N.J., Kaul S. (2015). Ultrasound stimulates formation and release of vasoactive compounds in brain endothelial cells. Am. J. Physiol. Circ. Physiol..

[51] Osada T., Gu Y.H., Kanazawa M., Tsubota Y., Hawkins B.T., Spatz M., Milner R., del Zoppo G.J. (2011). Interendothelial claudin-5 expression depends on cerebral endothelial cell-matrix adhesion by β(1)-integrins. J. Cereb. Blood Flow Metab..

[52] Davis C.M., Zhang W.H., Allen E.M., Bah T.M., Shangraw R.E., Alkayed N.J. (2021). Soluble Epoxide Hydrolase Blockade after Stroke Onset Protects Normal but Not Diabetic Mice. Int. J. Mol. Sci..

