# Supplementary material for: Role of Endothelial STAT3 in Cerebrovascular Function and Protection from Ischemic Brain Injury
Source: Int J Mol Sci. 2022 Oct 12;23(20):12167. doi: 10.3390/ijms232012167 (PMC9602684; doi:10.3390/ijms232012167)
Supplement: Supplementary file 1 [file ijms-23-12167-s001.zip › ijms-1901017-supplementary.pdf]

International Journal of
Molecular Sciences

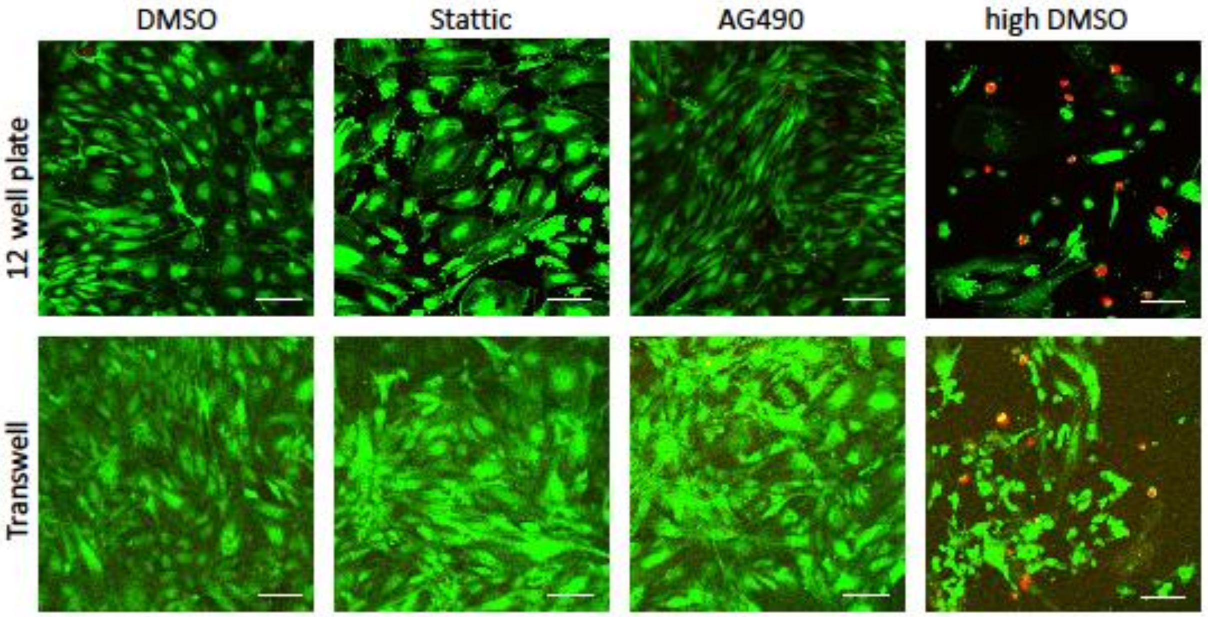

Figure S1. Calcein/PI labeling of ECs incubated for 12 h with 5 µM Stattic and 25 µM AG490 (12-well plate) or 1.25 µM Stattic and 12.5 µM AG490 (transwell) demonstrating that these concentrations are sub-lethal. High dose DMSO was used as a positive (cell death) control. Scale bar, 100 µm.
